# Integrated Analysis of Whole Genome and Epigenome Data Using Machine Learning Technology: Toward the Establishment of Precision Oncology

**DOI:** 10.3389/fonc.2021.666937

**Published:** 2021-05-12

**Authors:** Ken Asada, Syuzo Kaneko, Ken Takasawa, Hidenori Machino, Satoshi Takahashi, Norio Shinkai, Ryo Shimoyama, Masaaki Komatsu, Ryuji Hamamoto

**Affiliations:** ^1^ Cancer Translational Research Team, RIKEN Center for Advanced Intelligence Project, Tokyo, Japan; ^2^ Division of Medical AI Research and Development, National Cancer Center Research Institute, Tokyo, Japan; ^3^ Department of NCC Cancer Science, Graduate School of Medical and Dental Sciences, Tokyo Medical and Dental University, Tokyo, Japan

**Keywords:** artificial intelligence, whole genome analysis, epigenome analysis, machine learning, biomarker discovery, cancer diagnosis and treatment, precision oncology

## Abstract

With the completion of the International Human Genome Project, we have entered what is known as the post-genome era, and efforts to apply genomic information to medicine have become more active. In particular, with the announcement of the Precision Medicine Initiative by U.S. President Barack Obama in his State of the Union address at the beginning of 2015, “precision medicine,” which aims to divide patients and potential patients into subgroups with respect to disease susceptibility, has become the focus of worldwide attention. The field of oncology is also actively adopting the precision oncology approach, which is based on molecular profiling, such as genomic information, to select the appropriate treatment. However, the current precision oncology is dominated by a method called targeted-gene panel (TGP), which uses next-generation sequencing (NGS) to analyze a limited number of specific cancer-related genes and suggest optimal treatments, but this method causes the problem that the number of patients who benefit from it is limited. In order to steadily develop precision oncology, it is necessary to integrate and analyze more detailed omics data, such as whole genome data and epigenome data. On the other hand, with the advancement of analysis technologies such as NGS, the amount of data obtained by omics analysis has become enormous, and artificial intelligence (AI) technologies, mainly machine learning (ML) technologies, are being actively used to make more efficient and accurate predictions. In this review, we will focus on whole genome sequencing (WGS) analysis and epigenome analysis, introduce the latest results of omics analysis using ML technologies for the development of precision oncology, and discuss the future prospects.

## Introduction

**Figure 1:**
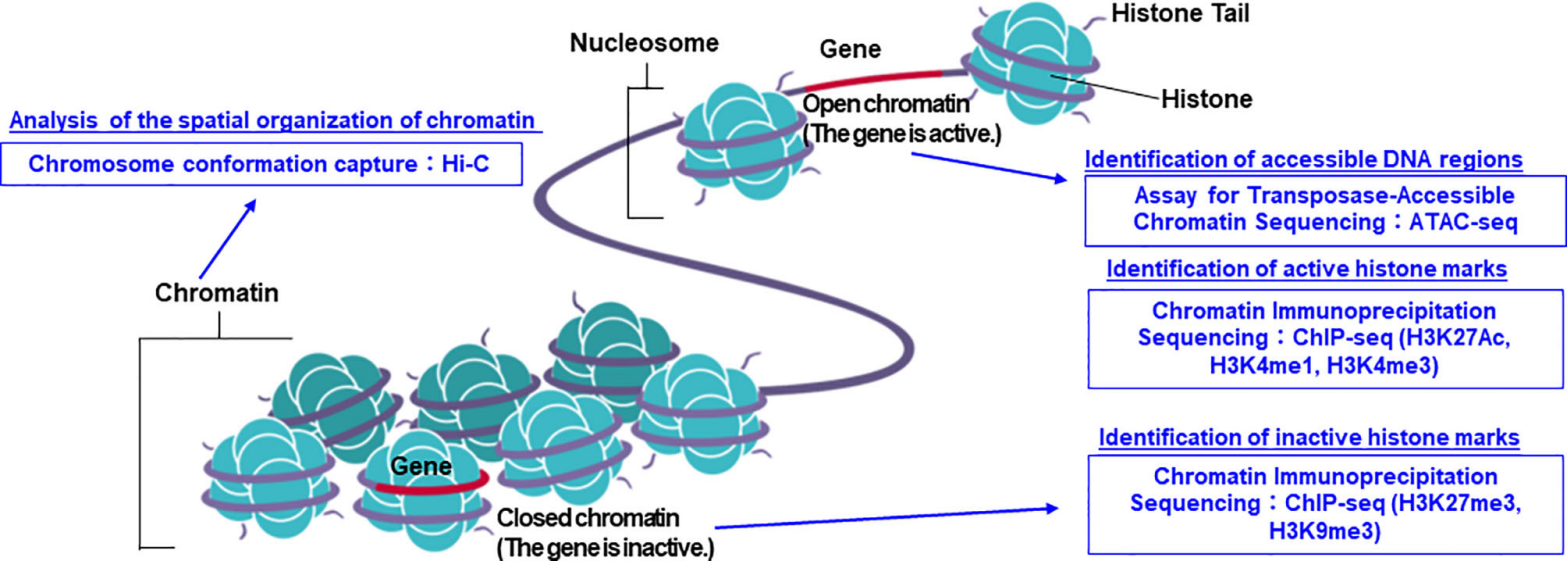
The summarized figure of chromatin structure and epigenomic analysis methods. ChIP-seq, ATAC-seq, and Hi-C methods can be used to predict the state of transcriptional activation or inactivation, and chromatin structure. Image credit: Shutterstock.com/ellepigrafica.

The structure of DNA was first reported by Watson and Crick in 1953 (1). Following this, the first sequencing technique known as the Sanger sequencing method was developed in 1977 (2). In 1987, the first automatic sequencing machine (AB370) was introduced by Applied Biosystems, which uses capillary electrophoresis without the need for a gel, which enabled the sequencing process to be more convenient in terms of accuracy and time (3). This technology truly accelerated the completion of the International Human Genome Project, which was aimed at decoding three billion human nucleotide base pairs (4). With the completion of the International Human Genome Project, the era known as the post-genome era began, and attempts to apply genomic information to medicine began to be actively pursued. Consequently, the concept of personalized medicine has also come to attract attention (5–7). Under such circumstances, the advent of a new analysis method called next-generation sequencing (NGS) technology has rapidly accelerated the speed of nucleotide sequence analysis and dramatically lowered the cost of performing whole genome analysis (8, 9). As a result, genome-wide analysis can now be performed routinely. In addition to DNA sequence analysis, various analysis methods using NGS technology have emerged, such as RNA sequencing (RNA-seq) for gene expression analysis, chromatin immunoprecipitation sequencing (ChIP-seq) for histone modification analysis and identification of transcription factor binding sites, Assay for Transposase-Accessible Chromatin using sequencing (ATAC-seq) and Hi-C for chromatin structure analysis (10, 11) (**Figure 1**). Along with technological innovation, there have also been attempts to apply genomic information to actual clinical practice. Targeted-gene panels (TGPs), which use NGS to examine the mutation status of a limited number of cancer-related genes, are actively being used to select the optimal treatment (12–14). On the other hand, one of the major problems in promoting precision oncology using the TGP method is that the number of patients who will benefit from the information obtained by the TGP method alone is limited (15–17). In order to increase the number of patients who will benefit from the promotion of precision oncology in the future, it is necessary to add more detailed omics data, such as whole genome analysis data and epigenome data, for integrated analysis. In recent years, it has been reported that epigenomic abnormalities play an important role in the development and progression of cancer (10, 18–25), and it is important to take into account information on epigenomic abnormalities when genomic mutations alone cannot elucidate the molecular mechanisms. In fact, the concept of epigenetic driver (epi-driver) is currently being used to describe the phenomenon of cancer development and progression based on epigenomic abnormalities (26, 27).

Another important issue is that the amount of data that researchers have to deal with has become enormous due to the emergence of various new methods with NGS analysis at their core as a result of technological innovation. For example, the amount of data generated by a single NGS run can be up to a million times larger than the data generated by a single Sanger sequencing run (28). In addition, there is a growing need for multimodal analysis, such as integrated analysis of genomic and epigenomic data, not just data from one modality. This kind of advanced analysis using a large amount of data is difficult to perform using conventional statistical methods, but nowadays, by proactively introducing artificial intelligence (AI) with machine learning (ML) and deep learning (DL) technologies at its core, good results can be obtained (29–31). In our view, there are four properties of ML and DL that are of particular importance. First, multimodal learning, which allows us to integrate multiple omics data as input (32–35). Second, multitask learning, which allows us to learn multiple different tasks simultaneously by sharing parts of the model (36, 37). Third, representation learning and semi-supervised learning, which allows us to acquire representations of data from large amounts of unlabeled data and thereby obtain small amounts of labels (38–41). The fourth is the ability to automatically acquire hierarchical features to capture higher-order correlations in the input (10, 42). More importantly, AI has already become one of the key technologies in the medical field, with a number of AI-powered medical devices approved by the US FDA (43). Under these circumstances, the active introduction of AI in the field of precision oncology seems to be an inevitable trend in the future.

Therefore, this review introduces the current status of efforts to establish precision oncology, focusing on whole genome sequencing (WGS) analysis and epigenome analysis, with particular emphasis on the results obtained through the use of ML and DL technologies.

## Whole Genome Analysis

In this section, we introduce the recently published up to date WGS analyses using ML and DL. The cost of WGS dropped from 100 million US dollars in 2001 to 1,000 dollars in 2020 (NIH National Human Genome Research Institute; https://www.genome.gov/about-genomics/fact-sheets/Sequencing-Human-Genome-cost; Cost per genome data - 2020). In 2020, an international collaboration to identify common mutation patterns in more than 2,600 cancer whole genomes was performed by the Cancer Genome Atlas Research Network as The Cancer Genome Atlas Pan-Cancer Analysis of Whole Genomes (PCAWG) project (44). The results described in the flagship paper were accompanied with related papers that focused on specific analysis, such as peak calls, structural variations (SV), and non-coding variants.

As summarized in **Table 1**, we categorized WGS analyses into five groups based on the purpose of their use. The first type of analysis considered is peak calling. Finding an accurate peak calling is one of the most important and difficult parts of WGS analysis. Aligning several hundred bps to the whole genome (three billion bps in length) while considering sequencing errors is technically challenging (65, 66). Thus, reports comparing the benchmarks and new pipelines, particularly deep neural networks (DNNs), have been published for both peak calling and the identification of variants (45–51) in **Table 1**. In general, DNN models were first trained with publicly available datasets followed by the evaluation of their performance with the test dataset. Validation is performed with the validation dataset either using publicly available data or their in-house dataset. For example, the WGS dataset obtained from the PCAWG was used for training and testing the model. To independently validate the DNN model, the authors assembled several datasets outside the PCAWG (67).

**Table 1:** Overview of whole genome analysis using machine learning.

Features	Pipeline name	Brief summary	Reference
Peak calling, mutational signature, or *de novo* assembly	HipSTR (Haplotype inference and phasing for short tandem repeat)	This method identifies *de novo* STRs; genotyping 1.6 million STRs in the human genome using HipSTR can be done in an average of 10 CPU hours per sample.	Nat. Methods (2017) (45)
	BayesTyper	This method performs genotyping of all types of variation (including SNPs, indels and complex structural variants) based on an input set of variants and read k-mer counts.	Nature (2017) (46)
	Genomiser	This method identifies pathogenic regulatory variants in non-coding regions.	Am. J. Hum. Genet (2016) (47).
	DeepVariant	This is a universal SNP and small-indel variant caller using deep neural networks, highlighting the benefits of using automated and generalizable techniques for variant calling.	Nat. Biotechnol (2018) (48).
	ARC (Artifact Removal by Classifier)	This is a supervised random forest model designed to distinguish true rare *de novo* variants (RDNVs) from genetic aberrations specific to lymphoblastoid cell lines (LCLs) or other types of artifacts, such as sequencing and mapping errors.	Cell (2019) (49)
	N/A	This method addresses the challenge of detecting the contribution of non-coding variants to disease using a deep learning-based framework that predicts the specific regulatory and detrimental effects of genetic variants.	Nat. Genet (2019) (50).
	NeuroSomatic	This is a convolutional neural network for somatic mutation detection.	Nat.Commun (2019) (51).
Genome graph	Graphtyper	This is an algorithm and software for discovering and genotyping sequence variation, which rearranges short read sequence data into a pan-genome and creates a graph structure that takes into account the mutations that encode sequence variation in a population by representing possible haplotypes as graph paths.	Nat. Genet (2017) (52).
	N/A	The results of the missing mutations are added to a structure that can be described as a mathematical graph, the genome graph. Compared to the existing reference genome map	bioRxiv (2017) (53)
		(GRCh38), the genome graph can significantly improve the percentage of reads that map uniquely and completely.	
	GenGraph	This provides a set of tools for generating graph-based representations of sets of sequences.	BMC Bioinformatics (2019) (54)
	N/A	This is a SV caller that uses genome graphs, which is used to analyze cancer somatic DNA rearrangements and revealed three novel complex rearrangement phenomena.	Cell (2020) (55)
Heterogeneity	PyClone	This is a Bayesian clustering method for grouping sets of deeply sequenced somatic mutations into putative clonal clusters while estimating their cellular prevalences and accounting for allelic imbalances introduced by segmental copy-number changes and normal-cell contamination.	Nat. Methods (2014) (56)
	MOBSTER	This is an approach for model-based tumor subclonal reconstructions. Cancer genomic data are generated from bulk samples composed of mixtures of cancer subpopulations, as well as normal cells. Subclonal reconstruction methods based on machine learning aim to separate those subpopulations in a sample and infer their evolutionary history.	Nat. Genet (2020) (57).
	DigiPico/MutLX	This method is a powerful framework for the identification of clone-specific variants with high accuracy.	ELife (2020) (58)
Mutational signature	SigMA (signature multivariant analysis)	This provides an accurate identification of mutational signatures with a likelihood approach, even when the mutation count is very small.	Nat. Genet (2019) (59).
	DeepMS (deep learning of mutational signature)	This is a regression-based model to estimate the correlation between signatures and clinical and demographical phenotypes in order to identify mutational signatures.	Oncogenes (2020) (60)
	SigLASSO	This method performs efficient cancer mutation signature analysis by accounting for sampling uncertainty, and also improves performance by allowing knowledge transfer through cooperative fitting of linear mixtures and maximizing sampling likelihood.	Nat. Commun (2020) (61).
GWAS	COMBI	This is a two-step algorithm that trains a support vector machine to determine candidate SNPs and then performs hypothesis testing on these SNPs.	Sci Rep (2016) (62).
	DeepWAS	This integrates regulatory effects predictions of single variants into a multivariate GWAS setting and provide evidence that DeepWAS results directly identify disease/trait-associated SNPs with a common effect on a specific chromatin feature.	PLoS Comput. Biol (2019) (63).
	Promoter-CNN + ALS-Net	This is a DL-based approach for genotype-phenotype association studies to predict the occurrence of ALS from individual genotype data. A two step-approach employs (1); promoter regions that are likely associated to ALS are identified and (2) individuals are classified based on their genotype in the selected genomic regions.	Bioinformatics (2019) (64)

The second analysis type is a genome graph or graph-based genome alignment. This approach has been recently reported and summarized (68). The advantage of using genome graphs is that they can accurately map (genotype) the polymorphisms of genomes with a good visualization, as well as perform fast and memory-efficient alignments (52–55) in **Table 1**. There is increasing recognition that a single, linear, monoploid reference genome is not always the best reference structure for human genetics, because they represent only a small fraction of existing human variations, particularly when they span SV breakpoints.

Third, heterogeneity in samples can be analyzed. Cancers are often observed to have various morphologies. These types of results are inconsistent with peak calls because they reflect where tissue samples are dissected. However, it is also true that tumors are composed of subpopulations of cells, and some cancer cells can migrate to other tissues. This heterogeneity results in a variety of features that can affect cancer phenotypes. To handle this, some published papers specifically focused on and investigated these phenotypes (56–58) in **Table 1**.

The fourth category is mutational signatures. The patterns of mutation or substitution signatures in cancer genome are discernible. Therefore, to categorize them, mutational signatures have been reported. Mutational signature analysis algorithms produce a decomposition matrix by using ML, a non-negative matrix factorization (NMF) approach, to extract mutational signatures (69–72). Additionally, other pipelines have been reported to perform mutational signature analyses to classify the samples (59–61) in **Table 1**.

The last is ML in a genome-wide association study (GWAS). GWAS has been used to discover genetic variants that are associated with diseases (73). To improve the analysis of GWAS, a combination of ML and DL analyses was reported (62) in **Table1**. However, how to improve mapping of regulatory variants (non-coding regions) identified by GWAS is still on going. Therefore, Arloth et al. developed DL-based approach and showed SNPs identified by DL were nominally significant in classical univariate GWAS analysis (63) in **Table 1**. They also identified disease/trait-relevant transcriptionally active genomic loci by integrating gene expression and DNA methylation quantitative trait loci (eQTL and meQTL) information of multiple resources and tissues. Although this is not a cancer research, another ML- and DL-based approach using GWAS data showed a good classification of amyotrophic lateral sclerosis (ALS) patient, and this approach can identify potentially ALS-associated promoter regions (64) in **Table 1**.

By integrating other omics data and analyzing single nucleotide variants (SNVs), indels, SV, and copy number alterations in non-coding regions, researchers can address the question of how pan-negative cancers developed, which we introduce in the following sections.

## DNA Methylation

DNA methylation is an epigenetic modification that can discriminate specific patterns between in normal tissue cells and in cancer cells (74, 75). These epigenetic alterations affect gene expression, and thus, cell-specific DNA methylation patterns are used in the diagnosis and treatment selection of cancer by identifying cancer-specific DNA methylation patterns in biopsy specimens and blood samples (76, 77). A few diagnostic measures utilizing cancer-specific DNA methylation patterns have already received FDA approval (78, 79). Moreover, ML and DL analyses have been increasingly used to identify novel disease-specific DNA methylation patterns; they have also been used in research that aims to utilize the DNA methylation data from cancer patients for diagnosis, staging, and prognosis predictions (80–83).

Cell-free DNA (cfDNA) is circulating DNA found in plasma, and is known to be elevated in cancer patients (84). The clinical significance of analyzing cfDNA is that (1) it is noninvasive (2), it can be applied for monitoring, and (3) it can detect a more global signature compared to the data obtained from a biopsy on a single metastatic site. Therefore, ML can be applied for DNA methylation analyses using cfDNA. The DNA methylation levels of plasma cfDNA in renal cell carcinoma (RCC) patients have been assessed by cell-free methylated DNA immunoprecipitation and high-throughput sequencing (cfMeDIP-seq), and RCC detection was performed using the elastic net regularized generalized linear model method (80). In this aforementioned study, DNA methylation data obtained from blood and urine samples were used for validation, and the area under the receiver operating characteristic (AUROC) curve was found to be of 0.99 for blood samples and 0.86 for urine samples, respectively. In another study, cfDNA methylation data from blood samples of patients with intracranial tumors were obtained with cfMeDIP-seq and successfully used to generate a cancer detection model using the Random Forest algorithm (81). This model was also shown to have high discriminative capacity among the five tumor types (isocitrate dehydrogenase (IDH) wild-type glioma, IDH mutant glioma, low-grade glial-neuronal, hemangiopericytoma, and meningioma).

Next, we review DNA methylation analyses that use solid tumor samples. First, to distinguish metastatic head and neck squamous cell carcinoma (HNSC) from primary squamous cell carcinoma of the lung (LUSC), DNA methylation data were extracted from surgical specimens of lung cancer patients and artificial neural networks (NN), and a support vector machine (SVM) and a random forest (RF) classifier was constructed because current diagnostics show no possibility to distinguish metastatic HNSC from primary LUSC. Authors developed models that classified 96.4% of the cases by NN, 95.7% by SVM, and 87.8% by RF (82). The DL-based approach is also used to detect DNA methylation patterns related to breast cancer metastases and predict recurrence by conducting feature selection using an autoencoder with a single hidden layer followed by ML techniques for classification, or enrichment analysis for finding a biological relevance, genomic context, and functional annotation of best genes (83).

## Cancer Epigenetics With A Focus On Enhancer Function

As mentioned earlier, since the advent of NGS technology and analyses based on ML, remarkable progress has been made in understanding the genetic basis of cancer. These studies have mainly defined genetic alterations as either causal (driver mutations), which confer a selective advantage to cancer cells, or consequential (passenger mutations, not directly causal), which do not have a selective advantage (26). Furthermore, genomic sequencing of tumor samples has revealed that different patients share a unique combination of one or two strong driver mutations such as gain-of-function EGFR and loss-of-function TP53 mutations typically detected in lung cancer and less frequent driver mutations (85, 86). On the other hand, the genetic component of the general disease risk is distributed mainly in the non-coding regions, which seem to be particularly rich in enhancers specific to the cell types associated with the disease (87, 88). Therefore, this has led to a growing interest in the annotation and understanding of human enhancers.

Measurable genome-wide biochemical annotations for enhancer regions include ChIP-seq or cleavage under targeted and release using nucleases (CUT&RUN) assays (89) for histone modifications or transcription factor (TF) binding, DNase I hypersensitivity sequencing (DNase-seq) for open chromatin (90), and ATAC-seq (91). On the other hand, it has long been hypothesized that enhancers loop in 3D space to access their target promoters. In recent years, the more powerful chromosome conformation capture (3C) method has yielded a series of high-resolution 3D conformation maps of the human genome in several cell types. In the 3C method, genomic DNA fragments are ligated to other genomic DNA fragments in physical proximity in the nucleus (92). These results have led to the identification of large compartments related to genomic organization, including enhancer-promoter loops (93), topologically associating domains (TADs) (94), and A/B compartments (92). In addition, 3C methods have been integrated with biochemical assays to annotate potentially functional interactions. For example, paired-end tag sequencing (ChIA-PET) (95), HiChIP (96), and proximity ligation-assisted ChIP-seq (PLAC-seq) (97) provide an overview of genome structures with a focus on proteins. Despite the development of various epigenomic methods as described above, and the obvious importance of human enhancers in both basic and disease biology, we still do not understand the repertoire of enhancers, including where they reside, how they act, and through which genes they mediate their effects.

**Figure 2:**
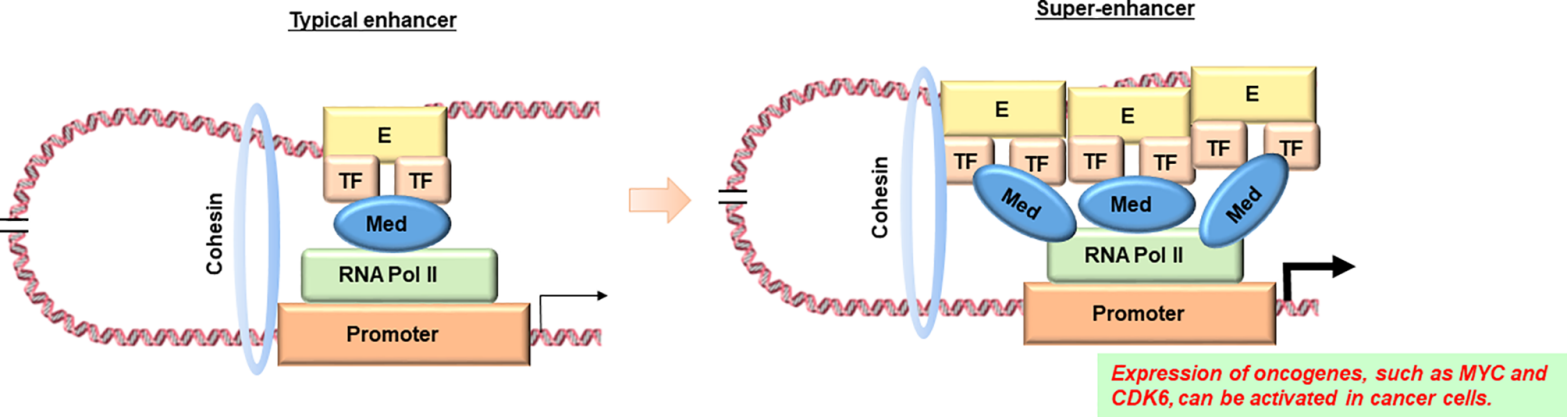
Diagram of comparison between a typical enhancer and a super-enhancer. According to reference 87, super enhancers are observed in the transcriptional regulatory regions of oncogenes such as *MYC* in cancer cells, but not in their counterparts in normal tissues. E, enhancer; TF, transcription factor; Med, Mediator complex; RNA pol II, RNA polymerase II. Image credit: Shutterstock.com/ellepigrafica.

In addition, it has recently been reported that super-enhancers are involved in abnormal gene expression in cancer cells (98). A super-enhancer is a region of the mammalian genome consisting of multiple enhancers, which are joined by a sequence of transcription factor proteins to drive the transcription of genes involved in cell identity (**Figure 2**) (99). An interesting finding is that disease-associated genetic mutations are particularly prevalent in super-enhancers of disease-associated cell types (100). Furthermore, cancer cells have been found to produce super-enhancers for oncogenes and other genes important in cancer development, suggesting that super-enhancers play an important role in human cell health and disease identity (100, 101). Importantly, super-enhancers are enriched in active chromatin marks such as H3K27ac and H3K4me3, while they are depleted in posed marks such as H3K27me3 (102). Therefore, epigenetic dysregulation may be involved in the production of super-enhancers in cancer cells. Since many disease-specific genetic variants are observed in super-enhancers, it seems to be pretty important to combine the information on genetic variants in non-coding regions obtained by WGS with the information on super enhancers based on epigenome data and analyze them in an integrated manner. As an example of super-enhancer analysis using ML, Gong et al. used two-dimensional lasso to improve the reproducibility of the Hi-C contact matrix and then classified the TAD boundaries based on the insulation score (103). The results showed that a higher TAD boundary insulation score was associated with higher CTCF levels, which may vary by cell type. They also showed that strong TAD boundaries and super-enhancer elements frequently overlap in cancer patients, suggesting that super-enhancer insulated by strong TAD boundaries may be used by cancer cells as a functional unit to promote tumorigenesis (103). Furthermore, Bu et al. proposed a new computational method, DEEPSEN, for super-enhancer prediction using a convolutional neural network, which is a DL algorithm (104). The proposed method integrates 36 different features and shows that it is capable of genome-wide prediction of super enhancers compared to existing methods.

In transcriptome and epigenome profiling, one of the conservative ML approaches of cluster analysis often yields reproducible regulatory subtypes. In this way, somatic mutations in cancer, although chaotic, often converge in a regulatory manner. These events suggest that cancer cells follow the same rules of transcriptional regulation as normal cells, despite the presence of aberrant combinations of transcription factors and genomic enhancers (105). Furthermore, a major unresolved question is how primary cancer cells metastasize and what the molecular events underlying this process are. However, extensive sequencing studies have shown that mutations may not be the causative factors in the transition from primary to metastasis (106). On the other hand, epigenetic changes are dynamic in nature and may play an important role in determining the metastatic phenotype, and research in this area is only beginning to be evaluated (107, 108). Unlike genetic studies, the current limitations in studying epigenetic events in cancer metastasis are the lack of conceptual understanding and the lack of an analytical framework to identify the putative driver and passenger epigenetic changes. We would therefore like to introduce an ML analysis that has the potential to address these issues.

## Challenges That Machine Learning Can Overcome

Genomic and epigenetic data-driven science operates by comprehensively exploring genome-wide data to discover new properties, rather than testing existing models and hypotheses (109). These data-driven approaches include finding relationships between genotypes and phenotypes, searching for biomarkers for personalized medicine, discovering driver genes and predicting their functions, and tracking genomic regions with biochemical activities such as transcriptional enhancers, as mentioned in the previous section. Due to the large scale and complexity of genomic and epigenetic data, it is often not sufficient to check pairwise correlations to make predictions. Therefore, analytical tools are needed to support the discovery of new relationships, the derivation of new hypotheses and models, and to make predictions. ML is designed to automatically detect patterns in data, unlike algorithms that have predetermined assumptions and expertise. Therefore, ML is well suited for data-driven science, especially genomics and epigenomics (110). However, the performance of ML is highly dependent on how the data are represented and how each variable or a feature is extracted. Epigenetic information and various modalities are known to be interrelated events, which are thought to interact with each other to change gene activity patterns. Based on these hypotheses, Wang et al. predicted the DNA methylation state of a specific region using a deterministic ML model [stacked denoising autoencoders (SdAs)] based on the 3D genome topology and DNA sequence obtained from Hi-C experiments (111). Against the backdrop of the high cost and difficulty of experimental techniques, which is the bottleneck of Hi-C data acquisition, inference from 1D information such as ChIP-seq, ATAC-seq, and RNA-seq to 3D genome topology structure has been actively attempted using various ML methods (**Table 2**). However, the prediction accuracy may not be improved due to inaccurate extraction of the essential structures within the epigenetic dataset, such as the still unelucidated mechanism of gene transcription regulation by high-dimensional interactions between enhancer and promoter regions. To solve these issues, an integrated approach that combines not only the acquisition of multi-layered omics data over time but also the generation and selection of phenotypic features and ML, is necessary.

**Table 2:** Epigenetic analysis typically focusing on regulatory regions.

Features	Pipeline name	Brief summary	Reference
Epigenomic Atlas (chromatin marks/chromatin states, DHSs, active enhancers)	N/A	Mapping nine chromatin marks across nine cell types. Systematically characterizes regulatory elements, cell-type specificities, and functional interactions. Defining multicell activity profiles for chromatin state, gene expression, regulatory motif enrichment, and regulator expression. Assigning candidate regulatory functions to disease-associated variants from GWAS.	Nature (2011) (112)
	N/A	Presenting extensive map of human DNase I hypersensitive site (DHSs) to identify through genome-wide profiling in 125 diverse cells and tissue types. The map shows relationships between chromatin accessibility, transcription, DNA methylation, and mutation rate in regulatory DNA.	Nature (2012) (113)
	N/A	The bidirectional capped RNAs measured by cap analysis of gene expression (CAGE) are robust predictors of enhancer activity. Enhancers share properties with CpG-poor messenger RNA promoters but produce bidirectional, exosome-sensitive, relatively short unspliced RNAs. The generation of RNA is strongly related to enhancer activity.	Nature (2014) (114)
Regulatory sequence/Network identify (enhancer/promoter/EPI, *etc.*)	ELMER (Enhancer Linking by Methylation/Expression Relationships)	This uses methylation and expression data to identify cancer-specific regulatory transcription factors, detect enhancer-gene promoter pairs, and correlate enhancer status with expression of neighboring genes.	Genome Biol (2015) (115).
	JEME (joint effect of multiple enhancers)	This method is an inference of enhancer-target networks, and consists of two steps: identifying enhancers that regulate transcription start sites (TSSs) across all samples, and detecting enhancers that regulate TSSs in a particular sample, to determine the target genes of transcriptional enhancers in a particular cell or tissue.	Nat. Genet (2017) (116).
			
	FOCS (FDR-corrected OLS with Cross-validation and Shrinkage)	This method estimates the link between enhancers and promoters based on the correlation of activity patterns between samples and implements a leave-cell-type-out cross-validation (LCTO CV) procedure to avoid overfitting of the regression model to the training samples. The cross-validation scheme consists of learning training set of samples and evaluation left-out samples from other cell types. This also provides extensive enhancer–promoter maps from ENCODE, Roadmap Epigenomics, FANTOM5, and a new compendium of GRO-seq samples. FOCS suggests repressor–promoter links.	Genome Biol (2018) (117).
	SPEID (Sequence-based Promoter-Enhancer Interaction with Deep learning; pronounced “speed”)	This method predicts enhancer-promoter interactions using DL models from genomic sequences, using only the location of enhancers and promoters in specific cell types. Using the melanoma dataset, this shows that there is potential to identify somatic non-coding mutations that reduce or interrupt important enhancer-promoter interactions (EPIs).	Quant. Biol (2019) (118).
	EP2vec	This method uses natural language processing to predict enhancer-promoter interactions, and also extracts sequence-embedded features (fixed-length vector representations) using an unsupervised DL model, the paragraph vector. The extracted features are used to train a classifier to predict the interaction using supervised learning. This can also merge sequence embedded features with experimental features for more accurate prediction.	BMC Genomics (2018) (119)
Inference of the 3D structure of chromatin	Transcriptional decomposition	This separates RNA expression into positionally dependent (PD) component and positionally independent (PI) effects by transcriptional decomposition method to show the predictability of fine-scale chromatin interactions, chromosomal positioning, and three-dimensional chromatin architecture.	Nat. Commun (2018) (120).
	CHINN (Chromatin Interaction Neural Network)	This predicts chromatin interactions between open chromatin regions using DNA sequence and distance using convolutional neural network. This also extracts sequence features and feed into classifiers.	bioRxiv (2019) (121)
	HiC-Reg	This method uses one-dimensional regulatory signals (chromatin marks, architecture, transcription factor proteins, and chromatin accessibility) and the published Hi-C dataset as training count data to predict cell line-specific contact counts. A random forest regression model is used as the main prediction algorithm.	Nat. Commun (2019) (122).

## Integrated Analysis Of Whole Genome Sequencing And Epigenome Datasets

For decades, cancer genome research has made significant progresses in the identification of driver gene mutations, largely owing to the wide application of WES. However, we are now realizing that druggable gene mutations are limited, and the majority of cancer patients are left with unmet medical needs. Therefore, academic interest has gradually shifted to the analysis of mutations in non-coding genomes based on WGS analysis and the search for “epi-drivers”, which are mechanisms of cancer development and progression caused by epigenomic abnormalities. For this purpose, WGS and epigenetic sequence technologies such as ChIP-seq, ATAC-seq, and Hi-C are effective tools because they offer comprehensive information about the genome, epigenome, and crosstalk between these (**Figure 1**).

Integrated analysis of genome and epigenetic data can be applied to predict the functional significance of single nucleotide polymorphisms (SNPs) and germline/somatic mutations. In order to analyze the function of DNA mutations in non-coding genomes, it is important to focus on eQTLs, which are genomic sites involved in the variation of expression levels of target genes. It is known that most functionally active SNPs and mutations fall within the open chromatin region, especially at inferred transcription factor binding sites. Indeed, approximately 55% of eQTLs SNPs are reported to coincide with those of open chromatin-associated SNPs and mutations (123). An impressive study on integrated analyses of WGS, ATAC-seq, and RNA-seq datasets has been posted (124). In a case of bladder cancer, they found that a single base mutation in enhancer region of the *FGD4* gene generated a putative *de novo* binding site for an NKX transcription factor, associated with an increase in chromatin accessibility and *FGD4* gene expression (124). Since high expression of the *FGD4* gene correlates with worse clinical outcomes in bladder cancer patients, this non-coding mutation might contribute to the malignant transformation of the cells by altering chromatin structure, thereby upregulating *FGD4* gene expression.

However, it should be noted that the majority of non-coding mutations might not exert an active function. In general, the regional mutation rates of human cancer cells tend to be higher in repressive chromatin states than in active chromatin states, which may reflect differing efficiencies of DNA repair signals or mutagen exposure (125). Thus, from a probabilistic view, most of mutations in the heterochromatin region occur only because of their closed chromatin states; that is, they are less likely to have any selective advantages or active functions. Intriguingly, this tendency toward higher mutational occurrences in heterochromatin states offers potentially useful information. By applying the ML model, genome-wide mutation data can be utilized to infer the cell-of-origin of cancer cells. For example, the mutational landscape of melanoma is best correlated with the epigenetic profile of skin melanocytes than skin fibroblasts or skin keratinocytes, suggesting the true cell-of-origin of melanoma (126). This approach can be clinically applicable to predict the cell-of-origin for cancer of unknown primary origin and may yield a better phenotypic understanding of them. WGS can resolve non-coding SVs and CNVs. RNA-seq detects the expression levels of driver genes and aberrantly expressed genes caused by alternative promoter usage and exon skipping (127–130). The utility of an integrative, comprehensive approach, with WGS, RNA-seq, and DNA methylation, independently and in combination, has been reported (130). Comprehensive molecular tumor profiling comprising WGS, RNA-seq, and DNA methylation analyses identified pathogenic variants and provided therapy recommendations, which could accelerate the development of precision medications.

Overall, the genomic and epigenetic data of non-coding regions contain enormous, complex and interdependent information, and we believe that integrated analysis, effectively utilizing ML and DL technologies, is important to discover new drivers of human cancer.

## Discussion

The genetic variants or SNPs were refined by the international haplotype map (HapMap) project to create a haplotype map of genes and genetic variants that affect health and disease (131–133). This project was attempted to genotype one common SNP in every 5,000 bps. At that time, it was believed that more than 99.9% of DNA sequences between any two people were identical, suggesting that only less than 0.1% of the genetic variants affect health and disease (https://www.genome.gov/11511175/about-the-international-hapmap-project-fact-sheet). Nowadays, analyzing WGS data has identified a considerable number of the genomic variants. The international consortium embarked on the 1000 Genomes Project to find common human genetic variations by applying WGS to a diverse set of individuals from multiple populations. High-throughput sequencing technologies do facilitate WGS in terms of accuracy, cost, and time. Almost two decades after the completion of the Human Genome Project, we have already entered a new era of sequencing, which led to individual genomic information becoming analyzable data. In practical terms, WGS analysis is becoming cost-effective. In addition, there is a trend to apply WGS routinely in both basic sciences and clinical cancer care to help us better understand and identify potential therapeutic targets or predictive biomarkers.

Epigenetics analyses were also drastically and positively affected by NGS. Chromatin conformations analyzed by ChIP-seq, ATAC-seq, or Hi-C are known to be related to cancer phenotypes (124, 134). Epigenetic alterations of DNA methylation at promoter and enhancer regions that induce chromatin dysregulation are found in cancer (135, 136). NGS analysis can help resolve both genetic and epigenetic alterations, and we expect to reveal the mechanism of pan-negative cancers using these data. From this point of view, we further introduced enhancers as an important concept in precision oncology. The current understanding is that enhancers bind to cell type-specific transcription factors, associate with regions of open chromatin, and are flanked by histones with H3K27ac and/or H3K4me1 modifications. These enhancers interact with promoters in 3D space and are either potentially primed or activated. Despite their obvious importance in both basic biology and disease biology, much remains to be learned about the relationship between enhancers and chromatin higher-order structure, including the identification of enhancer regions, how enhancers work, and through which genes they mediate their effects. In the future, we hope that multimodal analysis of multidimensional omics data by effective use of ML and DL techniques may contribute to precision oncology by providing an integrated understanding of more detailed molecular mechanisms.

## Concluding Remarks

In this review, we first summarized the importance of genomic and epigenetic data and introduced the importance of omics data of interest in each section. Cancer is one of the leading causes of death worldwide, and molecular mechanisms remain unknown in certain cancers, which are categorized as pan-negative cancers. Multi-omics analyses by simply integrating omics data may encounter difficulties in identifying the mechanism causing cancer because none of the methodologies can address the comprehensive understanding underlying pan-negative cancers. Therefore, as we reviewed here, integrating multi-omics analysis with the assistance of ML is required for future cancer studies because each omics data is tightly linked to each other, and all omics data are associated with patient outcomes. Currently, there are high expectations for the development of medical AI, and it is expected that AI technology will be actively introduced in actual clinical practice in the future. On the other hand, medical AI research for clinical applications is currently focused on medical image analysis (137–144), and research on the introduction of AI to omics analysis such as whole genome analysis and epigenome analysis, as well as its clinical application, has not progressed sufficiently yet. In this regard, one of the problems associated with the widespread adoption of AI-based methodologies in omics analysis is that even though sequencing technology and other advanced analytics are increasingly being used in research and clinical practice, there is still a lot of confusion about the best protocols to adopt for analysis. For example, the RNA-seq pipeline is not sufficiently standardized, and the methodology relies heavily on the expertise and experience of a single research group/bioinformatics. As a result, in areas where uncertainty remains, the spread of AI-specific technologies may be delayed. We hope that this review will trigger the interest of more researchers in this field, and that the standardization of omics analysis will actively promote the adoption of AI and contribute to the establishment of the field of precision oncology in the future.

## Author Contributions

KA and RH contributed to the study concept, design, and are guarantor of integrity of the entire study. KA, SK, KT, HM, ST, NS, RS, MK, and RH all contributed to literature search, manuscript preparation, and manuscript editing. All authors contributed to the article and submitted version.

## Funding

This work was supported by JST CREST (Grant Number JPMJCR1689), JST AIP-PRISM (Grant Number JPMJCR18Y4), JSPS Grant-in-Aid for Scientific Research on Innovative Areas (Grant Number JP18H04908), and JSPS KAKENHI (Grant Number JP20K17982).

## Conflict of Interest

The authors declare that the research was conducted in the absence of any commercial or financial relationships that could be construed as a potential conflict of interest.
